# Fibroblast-derived small extracellular vesicles loaded with tacrolimus enhances dermal delivery and alleviates cytokine-overdriven skin inflammation

**DOI:** 10.1007/s13346-026-02049-1

**Published:** 2026-02-17

**Authors:** Praveena Nanthakumar, Jia-Xian Law, Shiow-Fern Ng

**Affiliations:** 1 Centre for Drug Delivery Technology and Vaccine, Faculty of Pharmacy, Universiti Kebangsaan Malaysia, 50300 Kuala Lumpur, Malaysia; 2 Department of Tissue Engineering and Regenerative Medicine, Faculty of Medicine, Hospital Canselor Tuanku Muhriz UKM, Jalan Yaacob Latif, Bandar Tun Razak, Cheras, 56000 Kuala Lumpur, Malaysia

**Keywords:** Small extracellular vesicles, Tacrolimus, Controlled delivery, Cytokine overdrive, Skin inflammation, Long covid

## Abstract

Long COVID has been increasingly linked to chronic inflammatory skin conditions driven by cytokine overproduction. Topical tacrolimus, a calcineurin inhibitor, is commonly used to manage such conditions due to its immunosuppressive properties. However, due to poor dermal penetration, tacrolimus oftens produce to suboptimal efficacy and adverse effects such as local irritation and burning sensation. Effective management of chronic inflammatory skin conditions linked to long COVID necessitates targeted, controlled drug delivery into deeper skin layers to modulate excessive cytokine production and attenuate localized inflammation. This study explores fibroblast-derived small extracellular vesicles (sEVs) as a new controlled delivery vehicle for tacrolimus. The sEVs were isolated using sucrose-cushioned density ultracentrifugation and characterized by TEM, NTA, Dot blot, and MicroBCA assay, confirming their successful isolation and purity. Tacrolimus was encapsulated into sEVs via sonication, with successful drug loading confirmed by morphological and physicochemical characterization. The resulting Tac-sEVs exhibited an encapsulation efficiency of 79.19% ± 0.01. Franz diffusion studies revealed a rapid initial release within the first 10 h, followed by sustained higher release over time. Tape-stripping demonstrated significantly deeper dermal penetration of tacrolimus loaded sEVs (Tac-sEVs) compared with commercial tacrolimus ointment and free drug. Both tacrolimus and Tac-sEVs downregulated IFN-γ, GCS-F, IL-2, and IL-4 expression, indicating potent suppression of SARS-CoV-2 spike glycoprotein-induced cytokine overproduction. PKH-26 fluorescence labelling confirmed efficient cellular uptake, while cytotoxicity assays (Alamar Blue, CCK-8) showed high cell viability for both formulations. In summary, these results position Tac-sEVs as a safe and promising therapeutic platform for cytokine-driven inflammatory skin diseases associated with long COVID, meriting further clinical investigation.

**Graphical Abstract:**

**Figure:**
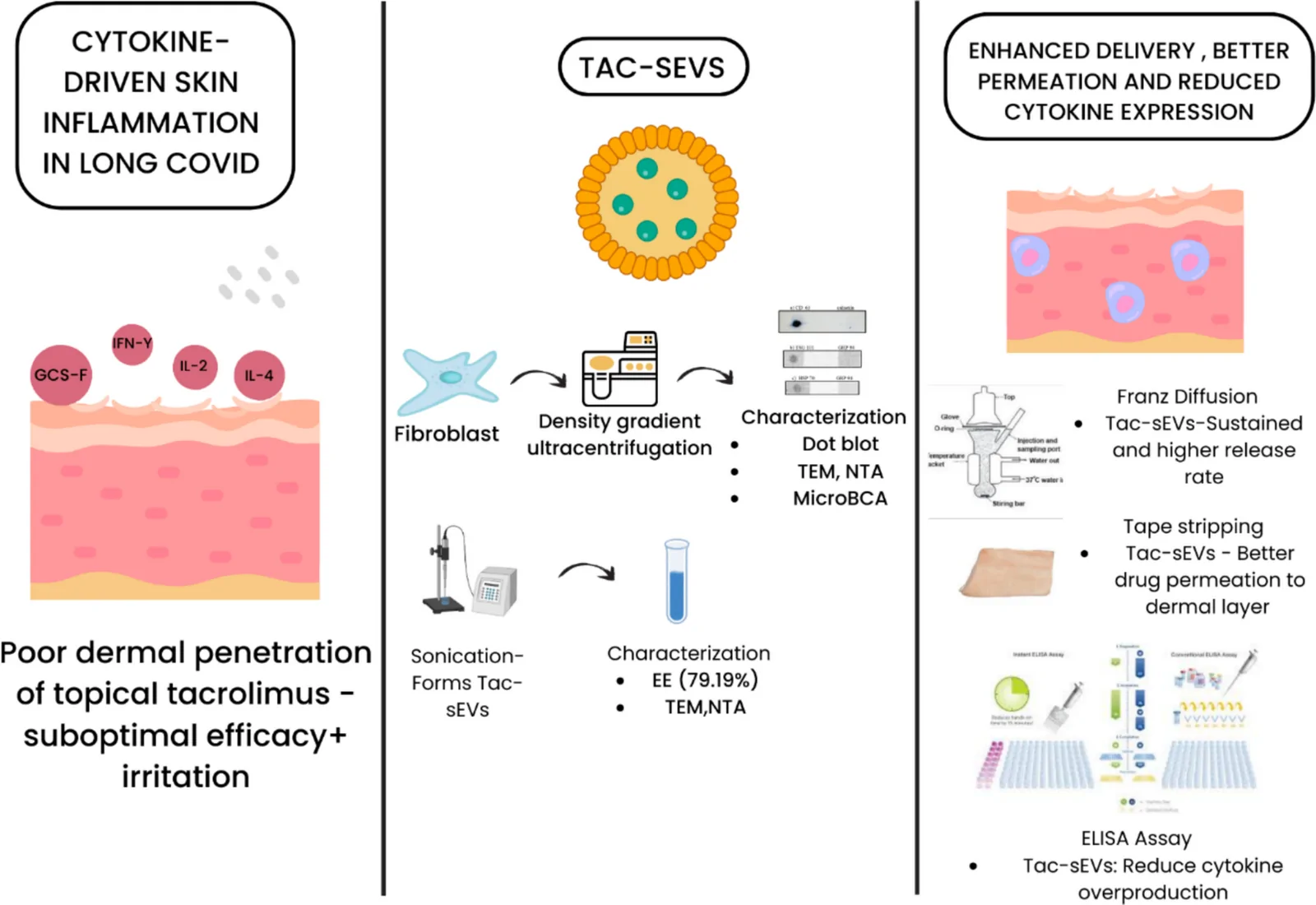

## Introduction

The global COVID-19 pandemic has brought unprecedented challenges to healthcare systems worldwide, not only due to acute viral infection but also because of a growing spectrum of persistent post-acute symptoms collectively referred to as “long COVID.” Among these manifestations, chronic skin inflammation has gained increasing attention as both a common and clinically significant complication. Cutaneous symptoms in long COVID often present as urticaria, chilblains, maculopapular rashes, and exacerbations of pre-existing inflammatory dermatoses such as psoriasis and atopic dermatitis [1, 2]. These conditions are frequently associated with sustained immune dysregulation and overproduction of pro-inflammatory cytokines which is a phenomenon sometimes referred to as a “cytokine storm.” In individuals with chronic inflammatory skin diseases, this cytokine-driven immune overactivation can lead to symptom exacerbation, extended flare durations, and increased difficulty in achieving disease control [3–5]. Cytokines such as IFN-γ, IL-2, IL-4, and granulocyte colony-stimulating factor (GCS-F) play central roles in driving cutaneous inflammation, perpetuating keratinocyte activation, immune cell recruitment, and barrier dysfunction [6, 7]. Thus, effective therapeutic strategies must not only address symptomatic relief but also directly modulate cytokine activity within affected skin tissues.

Tacrolimus, a macrolide calcineurin inhibitor, is widely used as a topical immunomodulator for inflammatory skin disorders. It acts by suppressing T-cell activation and reducing cytokine production, offering a steroid-sparing alternative in conditions like atopic dermatitis and psoriasis [8]. Despite its efficacy, tacrolimus faces significant delivery challenges. Its high molecular weight (822.05 Da) and lipophilicity (LogP ~ 3) hinder penetration through the stratum corneum, often causing the drug to remain trapped in surface lipids rather than reaching the inflamed dermis [9]. Conventional ointment-based tacrolimus formulations (0.03% and 0.1% tacrolimus) offer poor penetration and lack sustained release, leading to fluctuating concentrations. To compensate, higher doses are used, increasing risks of irritation, systemic absorption, and long-term safety concerns [10]. Consequently, there is an urgent need for advanced delivery systems that enhance dermal penetration and provide controlled release while minimizing systemic exposure and localized side effects.

Existing nanocarrier strategies for tacrolimus, including lipid-based carriers and polymeric micelles, aim to improve solubility and epidermal deposition. While these systems often outperform conventional ointments, they still face significant hurdles. Penetration beyond the stratum corneum remains modest, and enhanced surface deposition does not always ensure deep or uniform distribution [11–13]. Additionally, synthetic carriers can trigger irritation or immune responses and require complex optimization for stability [14, 15].

A promising alternative is the use of small extracellular vesicles (sEVs). These nano-sized (30–200 nm), naturally secreted lipid vesicles facilitate intercellular communication by carrying bioactive cargo [16]. Their biological origin provides high biocompatibility, low immunogenicity, and an innate ability to cross biological barriers [16]. For lipophilic drugs like tacrolimus, the sEV lipid bilayer offers an ideal hydrophobic environment for solubilization, while their nanoscale size allows for penetration into the deep dermis where inflammation is concentrated [17]. Crucially, sEVs possess unique biological advantages over conventional liposomes. They naturally display membrane proteins such as tetraspanins and integrins that facilitate active cellular uptake and tissue tropism [18]. While liposomes often require synthetic modifications to target specific cells or bypass complex barriers, sEVs utilize endogenous mechanisms like endocytosis to navigate biological environments [19].

Dermal fibroblasts are the primary stromal cells in the dermis, central to collagen synthesis and ECM remodelling [20]. Fibroblast-derived sEVs are chosen for their superior biological relevance to skin homeostasis and inflammatory modulation compared to more generalized MSC-derived sEVs [21]. This choice addresses an underexplored niche in dermatological research, leveraging their natural ability to enhance tissue repair. By sourcing sEVs from skin-relevant cells like fibroblasts, researchers can further enhance tissue targeting. Although research specifically on sEV-mediated tacrolimus delivery is emerging, their ability to protect cargo and penetrate deep skin layers makes them a superior platform for treating cutaneous diseases. To the best of our knowledge, this is the first study to engineer fibroblast-derived sEVs as a targeted nanocarrier for tacrolimus to treat cytokine-driven skin inflammation, including that associated with long COVID. Specifically, we aimed to (i) isolate and characterize sEVs and formulate tacrolimus-loaded sEVs (Tac-sEVs), (ii) evaluate Tac-sEVs in terms of particle size, morphology, encapsulation efficiency, and drug permeation, (iii) investigate their dermal pharmacokinetics and capacity to suppress cytokine production in vitro, and (iv) assess cellular uptake and cytotoxicity in human skin cells using in vitro assays. By integrating high-efficiency drug encapsulation, enhanced dermal penetration, and sustained release, our approach addresses the limitations of conventional tacrolimus formulations, offering a novel and versatile strategy for localized immunomodulation in dermatology with potential applicability across a range of inflammatory and immune-mediated disorders.

## Material and method

### Material

Human dermal fibroblast cells (HDF) was purchased from Lonza, Basel, Switzerland.HDF Cells were maintained and passaged using DMEM/Ham’s F-12, with L Glutamine; 500ML (DMEM-12-A), Trypsin–EDTA (0.25%) in HBSS (1x), with phenol red; 100 ml (TRY-3B), FBS Xtra Fetal bovine serum, collected in south America (FBS-16A) that was purchased from Capricorn Scientific, Germany and Pen-strep solution was obtained from Viva Cell Biosciences, Kansas City, US. Tacrolimus, Pharmaceutical Secondary Standard, PKH26 Red Fluorescent Cell Linker Kit for General Cell Membrane Labelling, Phosphate buffered saline, Sucrose, Tween 20 was obtained from Sigma-Aldrich, Germany. Anti-SARS-cov-2 spike glycoprotein s1antibody, Anti-CD63 antibody, Anti-Hsp70 antibody, Anti-TSG101 antibody, Anti-Calnexin antibody, Anti-GRP94 antibody was purchased from Abcam, Germany. Cell counting kit-8 was purchased from Dojindo, UK. Alcohol Absolute 99.7% (Denatured) was purchased from Chemiz, UK. Human Granulocyte Macrophage Colony Stimulating Factor (GM-CSF) Elisa kit, Human Interleukin 4(IL 4) Elisa kit, Human Interferon y(IFN-Y) Elisa kit, Human Interleukin 2(IL-2) Elisa kit was purchased from Bioswamp Life Sciences Lab, Wuhan, China. Micro BCA™ Protein Assay Kit, Alamar Blue™ Cell Viability Reagent, Pierce™ ECL Western Blotting Substrate, Deuterium oxide, for NMR, 99.8 atom % D was obtained from ThermoFisher Scientific, Massachusetts, USA. Porcine ears were obtained from local slaughterhouse located at Setapak, Malaysia. Mouse IgG Horseradish Peroxidase Conjugated Antibody were purchased from R&D Systems Minneapolis, USA. 10X Tris Buffered Saline (TBS), Ultra-Pure Grade was obtained from Vivantis Technologies, Selangor, Malaysia. Immobilon®-P Polyvinylidene difluoride membranes were purchased from Merck Millipore Ltd, County Cork, Ireland.

### Methodology

#### Isolation of small extracellular vesicles

##### Origin of sEVs, cell line and culture conditions

Small extracellular vesicles (sEVs) were derived from human dermal fibroblast (HDF) cells. Human dermal fibroblast cells (HDF) were cultured in cell culture media containing 10% Fetal Bovine Serum (FBS), 1% penicillin–streptomycin solution, 89% Dulbecco's Modified Eagle Medium/Nutrient Mixture F-12 (DMEM-F12) and expanded into 4 × T75 flasks until it reaches around 90% confluency.

##### Collection and pre-processing of conditioned medium for sEV isolation

Sucrose cushioned density ultracentrifugation technique was used to isolate sEVs from fibroblast cells as previously reported [22]. Then, the culture media was discarded and replaced with 20 ml of serum-free media for each flask. The flasks are then incubated at 37 °C, 5% CO2 for 48 h before the culture media supernatants (CMS) were collected. CMS were then transferred to an appropriate tube and centrifuged at 500 g for 5 min at 4°. Supernatant was collected and transferred to another tube and centrifuged again at 500 g for 5 min at 4° C. Pellet was discarded and the supernatant was transferred to another tube for centrifugation at 2000 g for 15 min at 4 °C. The pellet was discarded, and supernatant was collected and filtered through 0.22 µm. The filtrate then made up to 15 ml with PBS. For sucrose cushion preparation, 1.9 g of sucrose was weighed in a bijou tube very accurately. Then, the tube containing sucrose was left on the scale and deuterium oxide (D2O) was added to the tube containing sucrose until the weight reaches 7.6 g. The mixture was left to dissolve completely on a shaker at room temperature. Then sEVs isolation was done by filling up centrifuge tubes with 7.5 ml of precleared CMS. A layer of sucrose was added at the bottom of CMS by placing a glass pipette into the tube and 1 ml of 25% sucrose solution was added through the glass pipette. The tubes containing sucrose-CMS layers very carefully with minimal agitation were placed into buckets of the ultracentrifuge rotor. CMS were then ultracentrifuged at 100,000 g for 1.5 h at 4 °C. Upon completion, 650 µl were carefully pipetted out of the bottom sucrose layer and transferred into a tube containing 20 ml PBS. Sucrose layer was then filtered again through 0.22 µm and the filtered solution were transferred into a polycarbonate ultracentrifuge tube and ultracentrifuged at 100,000 g for 1.5 h at 4° C. Supernatant was discarded very carefully using a 5/10 ml pipette. Finally, the sEVs pellet was then resuspended in 400 µl PBS and then stored at 4° C.

#### Characterization of small extracellular vesicles

##### Morphological characterization

Transmission Electron Microscopy (TEM) was used for characterization of Small EVs and Tac-sEVs. Small EVs and Tac-sEVs samples were first prepared by fixing with glutaraldehyde (typically 2%) in phosphate buffered saline (PBS) to preserve their structure. Then the fixed samples were placed on carbon-coated copper grid and stained with phosphotungstic acid. The grid was then air-dried before imaging. The prepared grids were loaded into Transmission Electron Microscope. The resulting images provided information on sEV morphology.

##### Nanoparticle Tracking Analysis (NTA)

The sEVs and Tac-sEVs size distribution and particle concentration were determined using Nanoparticle Tracking Analysis (NTA). The samples were sent to Monash university (Malaysia) to determine the size distribution and concentration of sEVs and Tac-sEVs. Samples were diluted in particle-free phosphate-buffered saline to obtain particle concentrations within the optimal detection range of the instrument. Measurements were performed using automatic syringe injection at a constant flow rate. For each sample, multiple videos were recorded and analyzed using NanoSight software with identical camera level and detection threshold settings applied across all measurements.

##### Small EVs marker detection

The sEVs marker detection was performed using dot blot analysis to confirm the presence of sEVs in the sample as documented before and following manufacturer’s protocol [23]. Ten µl of sEVs in Phosphate-buffered saline (PBS) was spotted by using micropipette onto nitrocellulose membrane and dried with a stream of nitrogen. The step was repeated until a total of 40 µl of sEVs were spotted on the membrane. A 50 ml falcon tube was filled with 5 ml of 3% milk and the membrane spotted with sEVs was inserted into the tube. Then 3% milk was blocked for 1 h at RT on roller at a very slow speed. The milk was then discarded and the tube was filled up with 5 ml of fresh 3% milk in TBS. Five µl of primary antibody (1:1000) was added to the milk and the membrane was incubated in this primary antibody solution overnight at 4 °C. Primary antibody solution was discarded and the membrane was washed with 5 ml TBS-T on roller at RT for 5 min. TBS-T was then discarded and the washing step above was repeated twice more. A falcon tube was filled up with 3% milk in TBS-T and an appropriate volume of the secondary antibody was added. The membrane with secondary antibody solution was incubated for 1 h at RT on the roller. The secondary antibody solution was discarded and washed thrice with TBS-T as above. Detection was carried out by using Enhanced Chemiluminescence (ECL) system and membrane was imaged under Gel Doc for 15 min and 10 min. CD 63, HSP70 and TSG101 were positive markers that are used while calnexin and GRP94 were the negative markers used for the analysis.

##### Total protein quantification

Total protein concentration of sEVs was quantified by using the Micro BCA (Bicinchoninic Acid) Protein assay Kit following the manufacturer’s protocol. Nine standards were prepared by serial dilutions of the highest BSA standard which are 500, 250, 125, 62.5, 31.25, 15.625, 7.8125, 3.90625 and 0 µg/ml. Highest concentration of BSA standard (500 µg/ml) was prepared by taking 45 µl BSA stock and making it up to 180 µl with PBS. Eight eppendorf tubes were filled with 90 µl of PBS. 90µL from the highest BSA standard was taken and mixed into the 1 st tube then 90 µl was taken from this tube and mixed into 2nd Eppendorf tube. This step was repeated until the 7th tube and the 8th tube was just PBS (0 µg/ml). 40 µl of each standard was added into a well of 96-well plate and 2 replicates are made for each BSA standard. Next, samples, reagent mix, and assay preparation was made. 1:2 dilution of samples was made with PBS in a total volume of 90 µl (45 µl of sample, 45 µl of PBS). 40 µl of each sample was added into a well of 96-well plate and 2 replicates were made for each sample. Micro BCA reagent mix was prepared. 50 µl of MicroBCA reagent mix were added into each well and incubated at 37 °C for 30 min. The absorbance was measured at 562 nm on the plate reader. Standard curve was plotted from the absorbance readings, and the protein concentration was calculated from it.

#### Drug loading and characterization of Tac-sEVs

##### Preparation of Tacrolimus loaded small extracellular vesicles

Drug loading into sEVs was done by using sonication method, which facilitates the incorporation of the drug into the lipid bilayer of sEVs [24]. Purified 0.5 ml of sEVs were first mixed with tacrolimus in 1 mL of PBS (0.5 mg/ml). The tacrolimus–sEV mixture was transferred into sterile polypropylene microcentrifuge tubes and sonicated using a Model 505 Sonic Dismembrator equipped with a 0.25-inch probe tip. Sonication was performed at 20% amplitude for six cycles of 30’s on/30’s off (total sonication time of 3 min). To prevent overheating and preserve vesicle integrity, samples were kept on ice, with a 2-min cooling interval between cycles. Following sonication, the sEV–tacrolimus suspension was incubated at 37 °C for 60 min to facilitate recovery and resealing of the vesicle membrane.

##### Encapsulation efficiency

The calibration curve was plotted by preparing tacrolimus solution in PBS (as solvent) using UV- Visible spectrophotometer at different concentrations (0.5,0.4,0.3,0.2,0.1,0.08,0.06,0.04 mg/ml). The λ max (maximum absorbance) of tacrolimus was determined by screening 0.5 mg/ml drug solution over the entire UV range 200–700 nm. According to the result of screening, the λ max was observed at 203 nm. Encapsulation efficiency of Tac-sEVs was done by centrifuge the sonicated Tac-sEV at 13,000 rpm for 10 min in Amicon Ultra centrifugal filter with a 100 kDa molecular weight cut-off (MWCO) [25]. Following centrifugation, the amount of tacrolimus in the supernatant of the sample was estimated by reading the absorbance of the supernatant using UV- Visible spectrophotometer at 203 nm, corresponding the maximum absorbance peak of tacrolimus in solution. The percentage of encapsulation efficiency (EE%) was calculated using the following Eq. 1:

1 $$EE\%=\frac{Dt-Df}{Dt} x 100$$

where, Dt = Amount of total drug in sample.

Df = Amount of free drug in sample.

#### Cell proliferation and toxicity assay

##### Alamar blue assay

Alamar Blue Assay was used to assess cell proliferation and cytotoxicity after treatment with tacrolimus and Tac-sEVs following manufacturer’s protocol. The assay is based on the reduction of resazurin to resorufin by metabolically active cells, indicating cell viability. The cells were harvested in the log phase of growth and cells were counted using a hemacytometer when the cells in the flask are confluent. The cells were seeded in a 96-well plate at a cell density of 1 × 10^4^ cells per well in 100 µl complete media, so that 70–80% confluency was attained after 1 day. The seeded plate was kept in a CO2 incubator at 37 °C,5% CO2,95% humidity for 24 h to facilitate growth and adherence of cells to the plate surface. Each well was washed twice with 1 × PBS, and the cells were treated with Tacrolimus and tac-sEVs in 100 µl complete medium. Proper controls of blank medium, untreated seeded wells were included. The cells were incubated for another 24 h in an incubator. Alamar blue was added aseptically in an amount equal to 10% volume of the wells. It was then incubated for 2–4 h in a CO2 incubator. The optimum incubation time varies with cell type and seeding density. Cell toxicity and proliferation were determined by recording the absorbance at wavelength 570 nm and 600 nm after incubation. The complete media without cells, including Alamar blue, was considered blank. The ratio of treated and control cells were calculated in percentage using Eq. 2:

2 $$\% \;Cell \;Proliferation= \frac{\left(E2 \;x \;A1\right)-\left(E1\; x \;A2\right) }{\left(E2 \;x \;B1\right)-\left(E1\; x\; B2\right)} x 100$$

where,

E1 = Molar extinction coefficient (E) of oxidized Alamar blue at 570 nm, 80,586.

E2 = Molar extinction coefficient of oxidized Alamar blue at 600 nm, 117,216.

A1 = Absorbance of sample wells at 570 nm.

A2 = Absorbance of sample wells at 600 nm.

B1 = Growth control absorbance at 570 nm.

B2 = Growth control absorbance at 600 nm2.

##### Cell Counting Kit-8 Assay (CCK-8 Assay)

CCK-8 Assay was used to analyse the cell proliferation and cytotoxicity according to manufacturer’s protocol. Fibroblast cells were seeded in 24 well plate and pre-incubated for 24 h in the humidified incubator. 100 µL of Tacrolimus and Tac-sEVs were added respectively in the pre-incubated well plate (n = 3). Incubate the plate for 24 h in the incubator. 10 µl of CCK-8 solution was added to each well plate. The plate was incubated for another 4 h. The absorbance was measured at 450 nm using a microplate reader. The Eq. 3 below is used to calculate the survival rate.

3 $$Cell \;survival \;rate\; \% =\frac{\left( Asample-Ab \right)}{(Ac-Ab)}\;x \;100$$

#### Tac-sEVs in suppression of cytokine production

##### Production of Pro-inflammatory Cytokines with recombinant SARS-CoV-2 spike glycoprotein S1

The fibroblast cells were seeded 4.5 × 10^5^ cells per well in a 24 well plate. The cells were incubated in the CO2 incubator for 24 h for confluency. After confluency reached, 25 ug/ml of SARS-CoV-2 spike glycoprotein S1 antibody was added and incubated for 1 h. 100 µl of Tacrolimus and Tac-sEVs were added into well respectively (n = 3). After a 24 h of incubation, supernatant was taken out for ELISA assay.

##### Analysis of cytokine expressions

The collected supernatants were then analysed using the ELISA assay kit according to manufacturer’s protocol. For standard dilution preparation, 6 tubes were prepared with number 0–5 marked successively. 150 µl of standard diluent was added to each tube, then 150 µl standard was added to tube 1 and mixed. 150 µl solution was added from tube 1 to tube 2 and gently mixed. This procedure was repeated from tube 2 to tube 5. Tube 0 serves as a blank standard tube; therefore, each concentration of the tube is 1200 pg/ml, 600 pg/ml, 300 pg/ml, 150 pg/ml, 75 pg/ml, 0 pg/ml respectively. Wells were classified as standard wells, blank wells, and sample wells. 50 µl of each concentration standard was added to the standard wells, respectively with 2 parallel wells for each concentration. No samples were added to blank wells but 10 µl biotinylated antibodies were added. 40 µl of samples and 10 µl of biotinylated antibody was added to wells. The mixture was shaken and mixed gently without touching the wall. Then 50 µl of HRP- conjugated reagent was added to every well, except blank wells. Microplate was sealed and incubated at 37ºC for 30 min. Meanwhile, wash buffer preparation was done by dilute 30-fold wash buffer concentrate with 30-fold distilled water. The microplate was unsealed, liquid discarded, and microplate was dry pat with absorbent paper then 300 µl wash buffer was added to every well, waited for 30 s, liquid discarded, and the procedure was repeated 5 times and pat dry. Then 50 µl chromogen solution A and 50 µl chromogen B were added to every well, shake and mixed gently and incubated away from light for 10 min. After 10 min, 50 µl of stop solution was added to every well, the colour changed from blue to yellow that suggests the stop of the reactions. The well plate was measured using a microplate reader that measures absorbance at 450 nm. There are 4 types of interleukins measured which are IL-2, IL 4, IFN-γ and GCS-F.

#### Ex vivo studies

2.2.5 (a) was conducted to assess transdermal permeation of tacrolimus across porcine ear skin using static Franz diffusion cells by quantifying the cumulative amount of drug transported into the receptor compartment over time, whereas 2.2.5 (b) evaluated dermal pharmacokinetics using a tape-stripping approach to characterize the distribution and retention of tacrolimus within the stratum corneum, epidermis, and dermis.

##### Skin permeation study

Ex vivo skin permeation study was performed on porcine ear skin using static Franz diffusion cells. Porcine ears were collected from a local slaughterhouse which was located at Setapak, Kuala Lumpur wet market. The dorsal portion of porcine ear skin was shaved, and the subcutaneous fat was removed using blunt forceps. Skin was washed thrice with PBS (pH 7.4). The porcine skin samples of 2 cm^2^ diameter were punched and mounted between donor and receptor by clamp. The receptor compartment was filled with 5 ml of receptor medium consisting of PBS. Pure tacrolimus solution (control) and tac-sEVs equivalent to 0.5 mg/ml were filled in donor compartment. The whole assembly was maintained at 37 °C with the speed of rotation of the magnetic bead at 50 rpm. At specific time points, aliquots of 1.5 ml were withdrawn from the receptor compartment and replaced with equal volume of receptor medium to maintain sink conditions. The permeation study was conducted for a total duration of 24 h. The samples collected were analysed by UV visible spectrophotometer at 203 nm.

##### Dermal pharmacokinetics study

Ex vivo dermal pharmacokinetics was carried out by using tape stripping technique [26]. 19 mm Scotch™ (3 M, USA) cellophane tape was used for tape stripping. The tape stripping technique was performed on porcine ears. Pig ears were collected from a local slaughterhouse. Dorsal portion of pig ear skin was shaved, and the subcutaneous fat was removed using blunt forceps. The skin was washed thrice with PBS. A 2 cm piece of porcine skin was used. One set consisted of three individual pieces of skin. Controls (Tacrolimus solution, commercial tacrolimus ointment) and Tac-sEVs were applied separately to each piece of skin. The first tape strip was discarded as it may contain drugs in adhered part of formulations on skin surface. Then, 30 tape strips were taken to remove subcutaneous layers of the skin. The tape was then chopped into small pieces and soaked in 100% 5 ml of methanol for 24 h to extract drugs from the skin. The epidermis and dermis layer were separated by using scalpel. The separated skin layers were then also trimmed and soaked in methanol overnight to extract drugs penetrated in dermis and epidermis region respectively. The tape and skin layers were sonicated using a bath sonicator for 15 min before keeping for extraction for another 24 h. The samples collected were analysed by UV–visible spectrophotometer.

#### Cellular uptake by human dermal fibroblast cells

Paul Karl Horan 26 (PKH 26) was used as dye to label sEVs as instructed in manufacturer’s protocol. For the labelling of sEVs, based on MicroBCA assay, 20 µg of sEVs was quantified and diluted in 1 ml of diluent buffer. 3 µl of PKH26 was diluted in 1 ml of diluent buffer and mixed by pipetting. Diluted PKH 26 dye was added to diluted sEVs and mixed by pipetting. The mixture was incubated for 5 min in the dark at room temperature. 2 ml of 1% bovine serum albumin (BSA) was added in PBS to the tube with the dye and the sEV mix. centrifuged for 1 h at 110,000 × g at 4ºC. The supernatant was discarded, and the pellet was resuspended in 2 ml of PBS and centrifuged for another hour at 110,000 × g at 4ºC. The wash was repeated with PBS and resuspended the labelled sEVs. The Fibroblast cells were seeded in 6 well plates. 1 ml of media was added to the well plate. After the cells reached confluency the next day, media was changed and 100 µl of labelled sEVs were added. The labelled cell was viewed under an inverted fluorescence microscope (10X magnification) after 24 h. The unlabelled cells are used as control.

### Statistical analysis

All experiments were performed in triplicate (*n* = 3) unless otherwise stated. Data are presented as mean ± standard deviation (SD). Statistical analyses were conducted using GraphPad Prism software. For comparisons involving more than two groups, one-way or two-way analysis of variance (ANOVA) was applied as appropriate, followed by Tukey’s multiple comparison post hoc test. For comparisons between two groups, an unpaired Student’s *t*-test was used. Significant differences were considered statistically significant at *p* < 0.05.

## Result and discussion

### Characterization of small extracellular vesicles

#### Morphological characterization

**Fig. 1:**
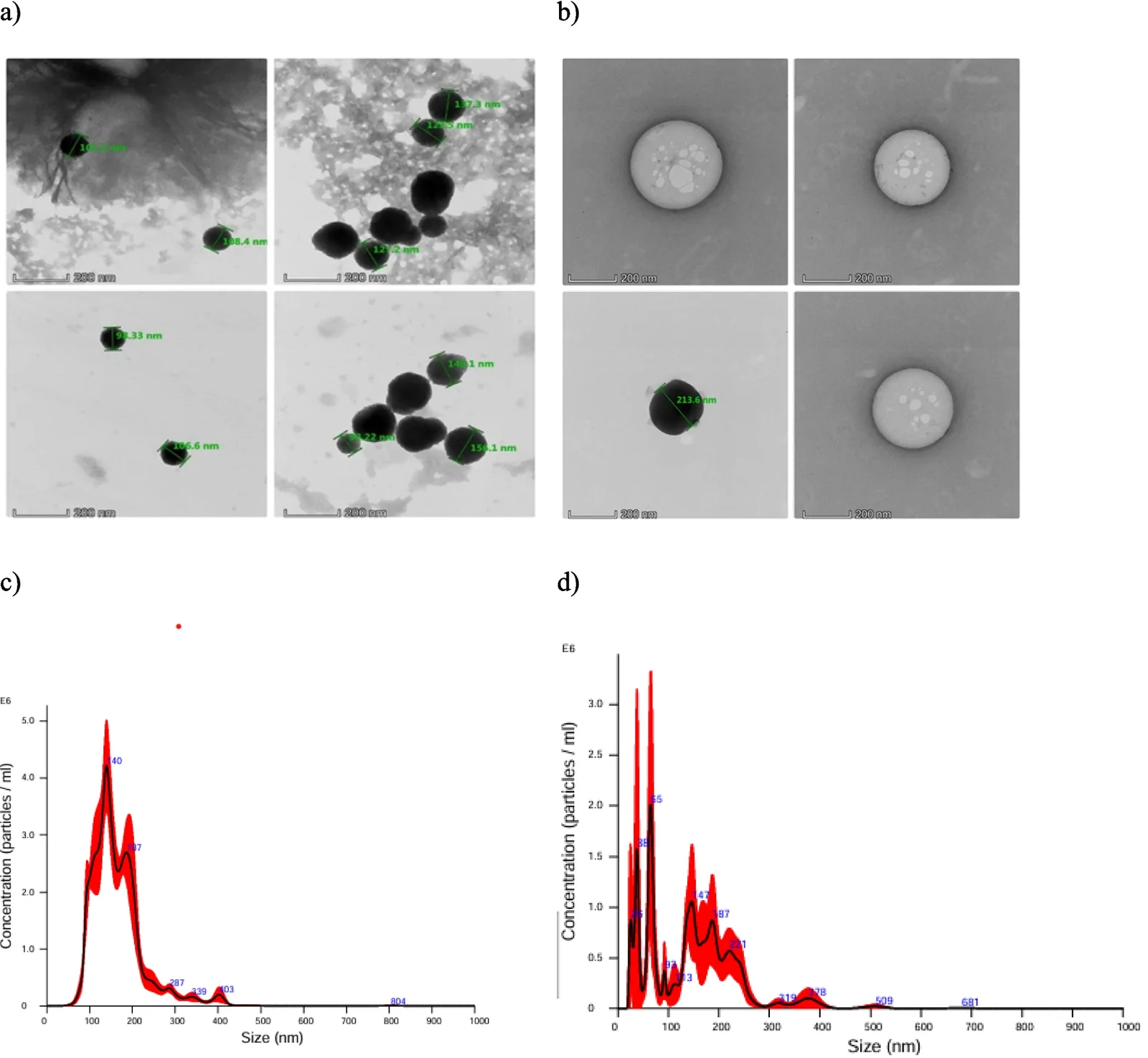
TEM images of sEVs(**a**) and Tac-sEVs(**b**) and NTA data of sEVs(**c**) and Tac-sEVs(**d**)

The TEM images of sEVs isolated from human dermal fibroblast cells as shown in Fig. 1(a), reveals several critical aspects regarding their morphology, size, and potential applications. The TEM images illustrate the spherical morphology typical of sEVs, which is a well-documented characteristic in the literature [27, 28]. The observed size range of these vesicles, approximately 90 nm to 190 nm in diameter, aligns with the established size range for sEVs (30–200 nm), thereby confirming their classification and distinguishing them from larger vesicles such as microvesicles [29]. The presence of larger sEV clusters or aggregates, particularly those exceeding 150 nm, may suggest incomplete dispersion during sample preparation, a phenomenon that has been previously reported in similar studies [27, 30]. The purity of isolated sEVs is crucial for downstream applications, as contaminants can interfere with functional assays and biomarker analyses [31]. The successful isolation of sEVs is further validated through nanoparticle tracking analysis (NTA) which provide additional insights into size distribution and concentration.

The transmission electron microscopy (TEM) images of drug loaded Tac-sEVs as shown in Fig. 1(b) reveal distinct spherical vesicles with intact and well-defined membranes. This morphology is characteristic of sEVs which are typically spherical and encapsulated by a lipid bilayer [32]. The preservation of the lipid bilayer structure during the loading process is crucial, as it indicates that the encapsulation of tacrolimus did not compromise the vesicle's integrity. This is significant because the stability of the vesicle is essential for its functionality as a drug carrier, ensuring that the encapsulated drug can be effectively delivered to target cells without degradationThe measured size of Tac-sEVs at approximately 213.6 nm is slightly above the typical size range for native sEVs, which is generally between 30 and 200 nm [33]. This increase in size is expected when drug molecules such as tacrolimus are loaded into the vesicles, as the additional mass can lead to a larger overall particle size. Nevertheless, the size remains within an acceptable range for drug delivery applications, as vesicles below 300 nm are known to facilitate cellular uptake via endocytosis [34]. The TEM images also exhibit internal granularity or structures within the tac sEVs, which indicate the successful encapsulation of tacrolimus. Given that tacrolimus is a hydrophobic drug, its interaction with the lipid bilayer or encapsulation within the vesicle lumen is a positive sign of effective loading. This encapsulation is critical for therapeutic efficacy of Tac-sEVs, as it ensures that the drug is protected from degradation and can be released in a controlled manner upon reaching target cells. The TEM images of Tac-sEVs revealed internal granularity and electron-dense structures, which may suggest successful encapsulation of tacrolimus within the vesicles. Similar internal contrasts have previously been reported for drug-loaded or cargo-enriched extracellular vesicles visualized by TEM [35].

#### Nanoparticle Tracking Analysis (NTA)

The characterization of sEVs through nanoparticle tracking analysis (NTA) reveals critical insights into their size distribution, concentration, and variability. Table 1 below displays key size distribution parameters (mean, mode, standard deviation, D10, D50, and D90) and particle concentration of sEVs and Tac-sEVs. The reported particle size distribution of sEVs predominantly falls between 100 and 300 nm, with a notable peak at approximately 166 nm, indicating a mean size of 166.5 nm and a mode of 131.8 nm. The D10, D50, and D90 values of 102.3 nm, 156 nm, and 239.6 nm, respectively, suggest that 10%, 50%, and 90% of the particles are below these sizes. This relatively narrow size distribution aligns with findings from other studies, which also report similar size ranges for sEVs, reinforcing the notion that sEVs typically exhibit a compact size profile suitable for various biological functions and therapeutic applications [32].

**Table 1:** Nanoparticle Tracking Analysis (NTA) data comparing the key size distribution parameters of small extracellular vesicles (sEVs) and Tacrolimus-loaded sEVs (Tac-sEVs)

Parameters	Small extracellular vesicles (sEVs)	Tacrolimus-loaded sEVs (Tac-sEVs)
Mean (nm)	166.5 ± 3.8	151.7 ± 28.9
Mode (nm)	131.8 ± 7.4	137.1 ± 37.3
Std deviation (nm)	63.8 ± 3.4	63.1 ± 10.9
D10 (nm)	102.3 ± 1.6	89.5 ± 24.3
D50 9 nm)	156.0 ± 6.3	140.4 ± 32.3
D90 (nm)	239.6 ± 10.3	228.1 ± 41.3
Concentration (Particle/ml)	3.99 × 108 ± 2.92 × 107	1.54 × 108 ± 3.81 × 107

The result for Tac-sEVs also indicate that encapsulated sEVs have a mean size of 151.7 nm with a standard deviation of 63.1 ± 10.9 nm. The mode is reported at 137.1 nm, which is significantly lower than the mean size, suggesting a presence of smaller vesicles within the sample. In contrast, native sEVs exhibit a mean size of 166.5 nm and a mode of 137.1 nm, indicating that they are generally larger than their drug-loaded counterparts. The D10, D50, and D90 values for Tac-sEVs (D50 = 140.4 nm) suggest a distribution centered around this median size, which is consistent with the expected size range for sEVs. The comparison with NTA result of sEVs reveals that tacrolimus loading results in a slight reduction in mean size compared to native sEVs. This reduction may be attributed to structural or compositional alterations induced by the drug loading process, potentially leading to the formation of smaller vesicles or a more compact structure [33]. The lower mode value in tacrolimus-loaded sEVs could indicate that the drug loading process has introduced variability in vesicle size, which is an important consideration for their application in drug delivery.

The concentration of particles in the analysed sample is reported as 3.99 × 108 particles/mL, with a standard deviation of 2.92 × 10^7^ particles/mL. This high concentration is advantageous for applications in diagnostics and therapeutics, as it allows for sufficient amount of vesicles to be utilized in downstream analyses and interventions. Previous studies have documented varying concentrations of sEVs, with some reporting concentrations as high as 2.4 × 10^9^ particles/mL in different biological contexts, indicating that the concentration of sEVs can be influenced by the source and isolation methods employed which is, in this study, isolated from urine samples by using modified exosome precipitation method [35]. The concentration of tacrolimus-loaded sEVs is measured at 1.54 × 10^8^ ± 3.81 × 10⁷ particles/mL, which is significantly lower than the concentration of native sEVs at 3.99 × 10^8^ ± 2.92 × 10^7^ particles/mL. The lower particle/frame count for Tac-sEVs (8.4 ± 2.1 particles/frame) compared to native sEVs (21.8 ± 1.6 particles/frame) further corroborates this finding. This reduction in concentration could be due to aggregation during the drug loading process or losses incurred during the isolation and purification steps. The implications of lower concentration are critical, as they may affect the therapeutic efficacy of Tac-sEVs, necessitating optimization of the loading and isolation processes to enhance yield [36]. The variability of concentrations may because of the extracellular vesicle release by cells is inherently variable, depending on cell state, culture conditions, and intrinsic biological heterogeneity. Even when the same cell line is used, independent isolation batches can yield different vesicle numbers and profiles because of biological fluctuations in EV biogenesis and secretion [37].

The standard deviation of sEVs, 63.8 nm and a standard error of 3.4 nm for the mean size indicate a consistent particle size within the sample, suggesting minimal variability. However, the mode size exhibits a slightly higher error of 7.4 nm, which may reflect some heterogeneity in the vesicle population. Such variability is not uncommon in sEVs studies, as the presence of different subpopulations of vesicles such as exosomes (typically 30 −150 nm in diameter), microvesicles (MVs) (generally 100—1000 nm in diameter), apoptotic bodies (usually 500 −5000 nm in diameter), can lead to variations in size and concentration [38]. The standard deviation for tacrolimus-loaded sEVs is reported at 63.1 nm, which is similar with observed for native sEVs. The observed reduction in mean size for tacrolimus-loaded sEVs is somewhat counterintuitive, as one might expect an increase in size due to the added mass of the drug. However, this phenomenon can be explained by several factors, including changes in surface tension or membrane curvature resulting from tacrolimus incorporation [39]. It is plausible that tacrolimus interacts with the lipid bilayer, potentially altering lipid packing and leading to a more compact vesicle structure rather than accumulating solely within the vesicle lumen. This interaction could be beneficial for maintaining the structural integrity of the vesicles while allowing for effective drug delivery.

NTA graphical representations (Fig. 1c) featuring merged data of sEVs with error bars, confirms the dominant size peak around 166 nm with minimal deviation, reinforcing the reliability of the NTA results. Such graphical interpretations are crucial for validating the reproducibility of measurements and ensuring that the data accurately reflect the characteristics of the sEVs being studied. Meanwhile, Fig. 1d data shows that Tac-sEVs are more polydispersed than native sEVs. This increased polydispersity indicates a more heterogeneous population in terms of size for the Tac-sEVs. Such variability may reflect differences in drug encapsulation efficiency among individual vesicles, where some vesicles may incorporate more tacrolimus than others, leading to size discrepancies. This heterogeneity could influence the pharmacokinetics and biodistribution of the drug-loaded vesicles, highlighting the need for careful characterization and potential strategies to enhance uniformity in future formulations.

#### Small EVs marker detection

**Fig. 2:**
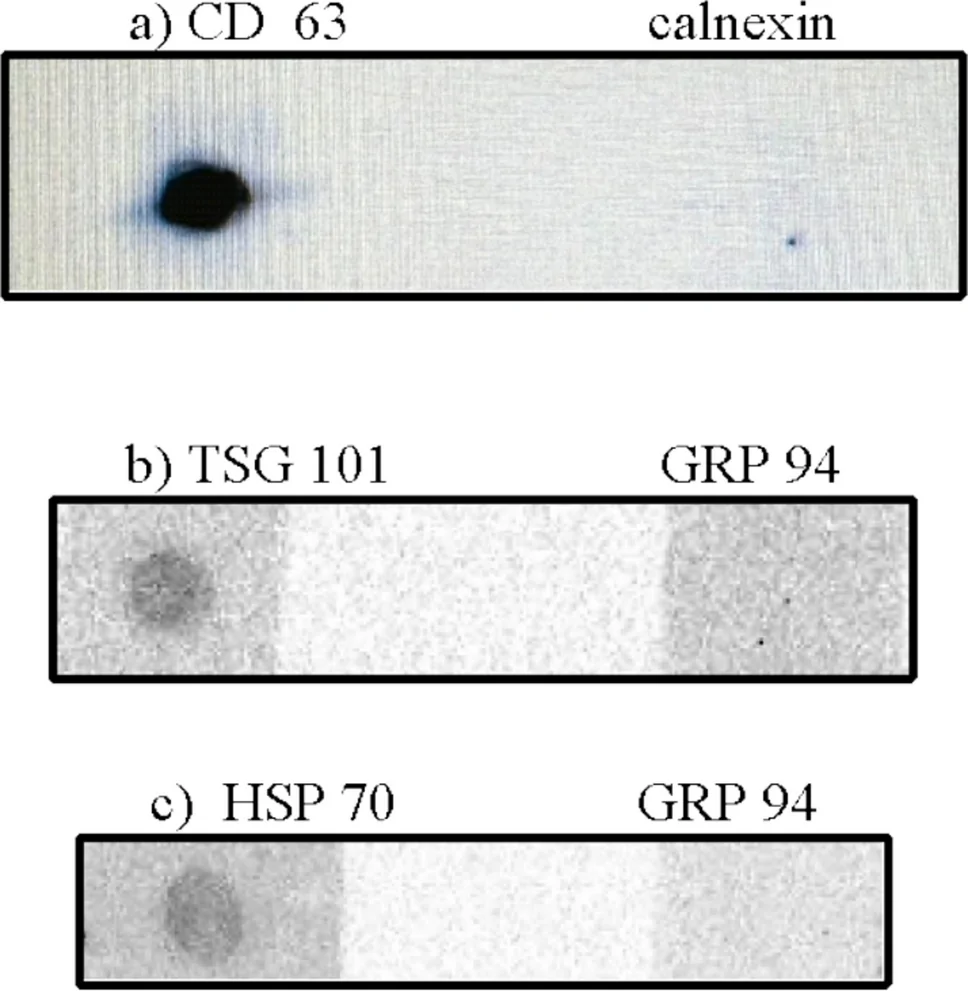
Characterisation of sEVs preparations by dot blot analysis. **a** CD 63 as positive marker and calnexin as negative marker, **b** TSG 101 as positive marker and GRP 94 as negative marker, HSP 70 as positive marker and GRP 94 as negative marker

Figure 2 below shows the result obtained from dot blot analysis for sEVs using CD63, TSG101, and HSP70 as positive markers, alongside GRP94 and calnexin as negative markers, provide significant insights into the characterization of these vesicles. The detection of signals for the positive markers and the absence of signals for the negative markers aligns with the established criteria for identifying sEVs including exosomes, as outlined in the Minimal Information for Studies of Extracellular Vesicles (MISEV) 2018 guidelines [40].

CD63 and TSG101 are well-recognized markers for sEVs, as they are associated with the endosomal sorting complex required for transport (ESCRT) pathway, which is crucial for the biogenesis of these vesicles [41]. The presence of HSP70, a heat shock protein, further supports the characterization of the vesicles as sEVs, as it is often found in the context of stress responses and has been implicated in the stability and function of extracellular vesicles [42]. The detection of these markers indicates that the vesicles isolated in this study are likely to be sEVs, which is consistent with findings from other studies that have utilized similar markers for validation [43]. Conversely, the lack of detectable signals for GRP94 and calnexin, both of which are markers associated with the endoplasmic reticulum, serves as a negative control and reinforces the specificity of the dot blot analysis [40]. The absence of these markers suggests that the isolated vesicles do not contain significant amounts of endoplasmic reticulum-derived proteins, which is a critical criterion for confirming the purity of sEVs, particularly exosomes preparations. This is particularly important as contamination with non-exosomal proteins can lead to misinterpretation of results and affect the functional studies of sEVs. The MISEV 2018 guidelines emphasize the importance of using a combination of positive and negative markers to accurately characterize extracellular vesicles [40]. The dot blot analysis provide compelling evidence that the isolated vesicles are indeed sEVs, characterized by the presence of established positive markers and the absence of negative markers. This aligns with the recommendations set forth by the MISEV 2018 guidelines and supports the reliability of dot blotting as a method for the characterization of small extracellular vesicles in various biological contexts [35].

#### Protein concentration determination

The quantification of sEVs using the Micro BCA assay yielded concentrations of 36.32 ng/µL, 32.28 ng/µL, and 21.91 ng/µL across three groups of sEVs. The sEVs samples were diluted 1:2 with the assay buffer prior to measurement, the measured concentrations were adjusted by a factor of 2 to account for this dilution. These results provide valuable insights into the yield and potential applications of the isolated vesicles, particularly in therapeutic and diagnostic contexts. The concentrations obtained in the study indicate a substantial yield of protein from the sEV preparations, which is crucial for downstream applications such as biomarker discovery, drug delivery, and therapeutic interventions. The observed protein concentrations are consistent with findings from other studies that have employed the Micro BCA assay for quantifying sEVs. For instance, Grishaev et al. reported similar methodologies for quantifying sEVs derived from cell lines, emphasizing the reliability of the Micro BCA assay in providing accurate protein content measurements [42]. This is particularly important as the protein content of sEVs can vary significantly depending on the source and isolation method, which can influence their biological functions and potential applications. Moreover, the variability in protein concentrations among the three groups suggests that the conditions under which they were isolated may have influenced the yield. This variability is not uncommon in sEVs studies, as different cell types and culture conditions can lead to differences in the quantity and composition of the vesicles produced.

Protein quantification is a fundamental component of extracellular vesicle characterization as it provides an estimate of vesicle-associated protein content, which is commonly used to assess EV yield, purity, and batch-to-batch consistency [38]. Measuring total protein helps normalize EV dosing in downstream functional assays and enables comparison between native and drug-loaded EV preparations. Additionally, the protein-to-particle ratio is often used as an indirect indicator of EV purity, where disproportionately high protein levels may suggest co-isolated soluble proteins or contaminants [44]. Therefore, protein determination complements particle size and concentration measurements and contributes to a more comprehensive and reliable evaluation of EV preparations.

#### Encapsulation efficiency of Tac-sEVs

The EE% of Tac-sEVs has been measured at 79.19 ± 0.01%. These results indicate a high level of efficiency in loading tacrolimus into the sEVs, which is crucial for their potential therapeutic applications. The EE is a critical parameter in the development of drug delivery systems, as it directly influences the dosage, efficacy, and safety of the therapeutic agents delivered. EE is defined as the percentage of the drug that is successfully loaded into the delivery vehicle compared to the total amount of drug used in the formulation process. High EE is desirable as it minimizes waste and maximizes the therapeutic potential of the drug. In the case of Tac-sEVs, the measured efficiencies of approximately 79.9% ± 0.002 suggest that a significant proportion of tacrolimus is retained within the vesicles. The encapsulation efficiencies observed for Tac-sEVs are comparable to those reported for other nanocarrier systems. For instance, Gómez et al. demonstrated that liposomal nanovesicles achieved encapsulation efficiencies of 82.7% and 84.1% for rifampin and teicoplanin, respectively [45]. These values indicate that while Tac-sEVs exhibit slightly lower encapsulation efficiencies, they still perform well within the context of drug delivery systems.

### Cell proliferation and toxicity assay

#### Alamar blue assay

The Alamar Blue assay results (Fig. 6a) indicate that both Tacrolimus and Tac-sEVs exhibit high levels of cell viability and proliferation, with proliferation rates of 90.86% and 90.83%, respectively. These findings suggest that both formulations maintain good cellular activity, which is crucial for their therapeutic potential in treating skin conditions. The similarity in proliferation rates indicates that neither formulation negatively impacts cell viability, suggesting that Tac-sEVs can deliver Tacrolimus effectively without altering its beneficial effects on skin cell proliferation. The comparable proliferation rates of Tacrolimus and Tac-sEVs are significant for several reasons. Firstly, the results demonstrate that Tac-sEVs, despite their enhanced penetration properties, do not compromise the biocompatibility of Tacrolimus. The ability of Tac-sEVs to deliver Tacrolimus without cytotoxicity give the potential of EV systems in enhancing drug delivery while preserving cellular integrity.

Tacrolimus, while effective, may lead to localized side effects if it remains concentrated in the upper skin layers. This is particularly relevant given the molecular weight of Tacrolimus (822 Da), which poses challenges for skin penetration. The retention of Tacrolimus in the stratum corneum can result in adverse effects, necessitating the development of better drug delivery systems that enhance deeper penetration into the dermis. Tac-sEVs have shown promise in this regard, as they can penetrate deeper into the dermal layer, thereby reducing the likelihood of localized side effects associated with Tacrolimus retention in the upper layers. The findings from the Alamar Blue assay suggesting that Tac-sEVs can serve as a safe and effective delivery system for Tacrolimus, enhancing its therapeutic potential while minimizing the risk of adverse effects. In conclusion, the Alamar Blue assay results provide compelling evidence that both Tacrolimus and Tac-sEVs exhibit high levels of cell viability and proliferation. The comparable proliferation rates suggest that Tac-sEVs can effectively deliver Tacrolimus without compromising its therapeutic effects. This research supports the continued exploration of Tac-sEVs as an innovative drug delivery system for Tacrolimus, with the potential to improve patient outcomes in the treatment of inflammatory skin conditions.

#### Cell counting kit-8 (CCK-8) assay

The CCK-8 assay is a widely utilized method for assessing cell viability and metabolic activity, providing insights into the effects of various compounds on cellular health. In the context of the results obtained from the CCK-8 assay as shown in Fig. 6b, the observations regarding Tacrolimus and Tac-sEVs reveal significant implications for their therapeutic potential. The cell survival rate (Fig. 6b) observed with Tacrolimus is 102.68%, indicating a slight enhancement in cell viability compared to the untreated control cells, which are set at 100% baseline. This finding suggests that Tacrolimus does not exert cytotoxic effects under the experimental conditions, thereby supporting its role as a non-toxic agent that can maintain or slightly enhance cellular activity. Previous studies have corroborated the non-cytotoxic nature of Tacrolimus, particularly in various cell types, Control Tacrolimus Tac-sEVs indicating its potential for therapeutic applications without adverse effects on cell viability.

In contrast, the cell survival rate for Tac-sEVs is markedly higher at 133.44%, indicating a robust enhancement of cell survival. This result suggests that Tac-sEVs not only maintain cell viability but also promote higher metabolic activity or proliferation compared to Tacrolimus alone. The results from the CCK-8 assay indicate that Tac-sEVs significantly enhance cell survival compared to Tacrolimus alone. This finding underscores the superiority of Tac-sEVs as a drug delivery platform, combining the therapeutic efficacy of Tacrolimus with the added benefits of sEV-mediated delivery. The implications of these findings are profound, suggesting that Tac-sEVs could be pivotal in applications requiring enhanced skin regeneration, repair, or targeted drug delivery. Future research should focus on elucidating the mechanisms underlying the enhanced efficacy of Tac-sEVs and exploring their potential in clinical settings.

In conclusion, both Alamar blue assay and CCK-8 assay confirms that both Tac-sEVs exhibit high level of cell viability, proliferation and significantly enhance cell survival rate. This in vitro data will be a crucial to further study the safety evaluation and biodistribution of Tac-sEVs in vivo [46].

### Tac-sEVs in suppression of cytokine production

**Fig. 3:**
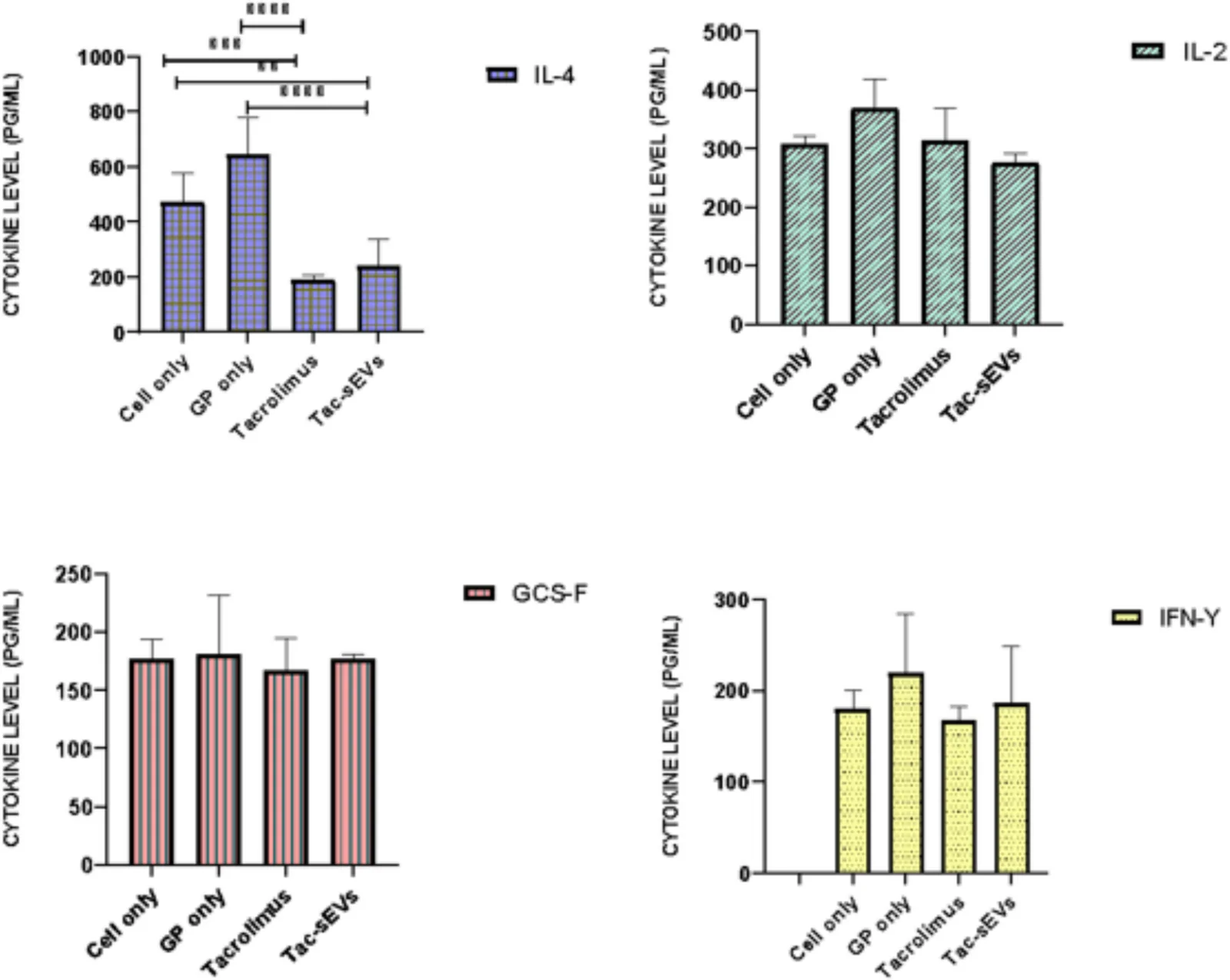
Cytokine expressions of Interferon gamma (IFN-Y), granulocyte-colony stimulating factor (GCS-F), Interleukin-2 (IL-2) and Interleukin 4 (IL-4) on untreated fibroblast cells, SARS-CoV-2 spike glycoprotein induced cells, treated with tacrolimus cells and treated with Tacrolimus loaded small extracellular vesicles (Tac-sEVs) cells; * represents 0.01 < *p* < 0.1; *n*=3

Interleukin-4 (IL-4) is the key cytokine in skin inflammation, with significant implications for the pathogenesis and treatment of conditions such as atopic dermatitis and other inflammatory skin disorders. IL-4 is primarily produced by Th2 cells and is pivotal in promoting a type 2 immune response, characterized by the recruitment of eosinophils, basophils, and mast cells to inflamed tissues, leading to chronic inflammation and barrier dysfunction. Studies have shown that overexpression of IL-4 can alter skin homeostasis, resulting in increased mast cell numbers and disturbances in collagen deposition, suggesting underlying pathological changes mediated by immunological modifications [47]. The ELISA data in Fig. 3 on IL-4 cytokine expression provides insights into the inflammatory response of skin cells under different conditions which are Cell only group (Baseline cytokine levels), GP only group (Cells treated with SARS CoV-2 glycoprotein), Tacrolimus only and Tac-sEVs group. The highest IL-4 expression is observed in GP only group, which significantly elevated reaching approximately 800 pg/mL compared to the baseline level of 500 pg/mL in untreated cells. For IL-4, GP only treatment resulted in a marked increase in cytokine production compared with the cell-only control. This elevation was highly statistically significant (*p* < 0.0001). Both free tacrolimus and Tac-sEVs significantly reduced IL-4 levels relative to the GP-only group. The reduction observed with free tacrolimus was highly significant (0.0001 ≤ *p* ≤ 0.001), while Tac-sEVs also produced a statistically significant decrease (0.01 < *p* < 0.1). No statistically significant difference was observed between free tacrolimus and Tac-sEVs (*p* > 0.1), indicating comparable efficacy in suppressing IL-4 production. This increase indicates that SARS-CoV-2 glycoprotein triggers a strong inflammatory response. This supports the fact that viral proteins can activate the Th2 immune pathway, lead to an increase in IL-4, a cytokine known to drive skin inflammation, allergic responses, and immune cell recruitment [48]. Among the treatment groups, Tacrolimus alone shows the most significant IL-4 suppression, with Tac-sEVs also reducing IL-4 levels but to a lesser extent. This suggests that tacrolimus effectively inhibits IL-4 production, likely due to its mechanism as a calcineurin inhibitor, which prevents NFAT-mediated cytokine transcription. Tac-sEVs reduce IL-4 but may have a slower effect, possibly due to a gradual release profile that does not achieve the same immediate suppression as free tacrolimus. The difference in suppression could be due to cellular uptake efficiency, drug bioavailability or delayed tacrolimus release from sEVs, leading to a lower immediate impact on IL-4 levels compared to free tacrolimus. IL-4 is a key driver of Th2-mediated inflammation, playing a central role in atopic dermatitis (eczema), psoriasis, and chronic skin inflammatory by increase immune cell recruitment, disrupt skin barrier function and promote chronic itch and inflammation. By reducing IL-4 levels, Tacrolimus directly mitigates Th2-driven inflammation, make it an effective anti-inflammatory therapy. Although Tac-sEVs also show suppression, their slightly lower efficacy suggests that further formulation optimization may be needed to enhance drug release or cellular targeting. However, Tac-sEVs still provides suppression and may offer advantages in prolonged drug retention and controlled release, which could be beneficial for long term inflammation management.

In psoriasis, a chronic inflammatory skin condition, IL-2 production is predominantly linked to the activation of T-cells, particularly Th1 and Th17 cells, which drive lesion development and maintenance. Research indicates that elevated levels of IL-2 contribute to the proliferation and activation of these Th1/Th17 cells, exacerbating inflammatory skin responses [49]. Unlike IL-4, which showed significant differences in expression levels, IL-2 levels remain relatively stable across all groups (Fig. 3), no statistically significant differences were observed among the groups (*p* > 0.1). SARS-CoV-2 glycoprotein (GP only) does not strongly elevate IL-2 levels compared to the control. This suggests that viral glycoprotein stimulation predominantly activates Th2 pathways (elevating IL-4) rather than triggering a strong Th1 response (which would elevate IL 2). Tacrolimus and Tac-sEVs do not significantly reduce IL-2 expression, implying that IL-2 is not a primary target of Tacrolimus-mediated immunosuppression in this model. Nevertheless, Tac-sEVs show a slight but non-significant reduction in IL-2 levels compared to Tacrolimus, indicating that drug release from sEVs might be slower or less effective in targeting IL-2 pathways. Unlike IL-4, which was significantly reduced by Tacrolimus, IL-2 remains largely unaffected by both Tacrolimus and Tac-sEVs. This suggests that Tacrolimus primarily targets Th2-driven inflammation (IL-4) rather than Th1/Th17 pathways (IL-2). Tac-sEVs may require further optimization to enhance drug delivery efficiency and intracellular release for broader cytokine modulation. IL-2’s regulatory role in immune homeostasis suggests that long-term effects of Tacrolimus and Tac-sEVs on T-regulatory cell function should be investigated.

Granulocyte Colony-Stimulating Factor (GCS-F) serves as a pivotal cytokine in the orchestration of neutrophil dynamics, influencing their production, survival, and recruitment, which are crucial processes in the innate immune response and various inflammatory skin conditions. Elevated levels of GCS-F are correlated with clinical manifestations in conditions such as psoriasis, neutrophilic dermatoses, and severe infections, wherein an overabundance of neutrophils contributes to tissue damage and chronic inflammation [50]. The ELISA data demonstrates as in Fig. 3 below shows that GCS-F expression remains unchanged across various experimental conditions, comprising the Cell only (Baseline control), GP only (SARS-CoV-2 glycoprotein stimulation), Tacrolimus treatment, and Tac-sEVs treatment groups. Statistical comparison confirmed that no significant differences were detected (*p* > 0.1), indicating that neither free tacrolimus nor Tac-sEVs substantially influenced GCS-F production in this model. This may infer that SARS-CoV-2 glycoprotein does not significantly upregulate GCS-F, indicating that viral exposure alone is insufficient to provoke neutrophilic inflammation. Additionally, Tacrolimus and Tac-sEVs do not exhibit suppressive effects on GCS-F, suggesting their anti-inflammatory mechanisms do not involve direct inhibition of neutrophil activity. The immune response observed in this model is likely more aligned with Th2-driven mechanisms, as suggested by the modulation of IL-4, further explaining the stable GCS-F levels throughout the experiments. Given that Tacrolimus and Tac-sEVs do not influence GCS-F expression, it is plausible that their therapeutic efficacy is primarily confined to Th2-mediated skin conditions (such as eczema and atopic dermatitis) rather than neutrophil-dominant disorders like psoriasis or bacterial skin infections.

The role of Interferon-gamma (IFN-γ) in skin inflammation is multifaceted, primarily associated with the modulation of immune responses orchestrated by T helper 1 (Th1) cells and natural killer (NK) cells. As a major pro-inflammatory cytokine, IFN γ is pivotal in the activation of macrophages, which enhances antigen presentation and recruitment of additional immune cells to sites of inflammation. Numerous studies have illustrated that excessive production of IFN-γ correlates with the pathogenesis of various autoimmune and inflammatory skin diseases, including psoriasis and vitiligo, by promoting keratinocyte hyperproliferation and impairing skin barrier function [51]. In the context of therapeutic interventions, experimental data on Tacrolimus and Tac-sEVs groups reveal interesting insights. Figure 3 indicated a decrease in IFN-γ expression when comparing treatment groups (i.e., Tacrolimus and Tac-sEVs) to untreated controls stimulated by SARS-CoV 2 glycoproteins. This suggests that these treatments have some capacity to modulate Th1 responses; however, the reduction in IFN-γ levels was not statistically significant. This outcome indicates that while there may be a trend towards suppression of Th1 mediated inflammation, Tacrolimus and Tac-sEVs primarily act on Th2 pathways, which are more pronounced in inflammatory skin conditions like atopic dermatitis [52, 53]. The implications of these findings are notable, particularly concerning the therapeutic applications of Tacrolimus and Tac-sEVs in skin inflammatory conditions. Tacrolimus has established efficacy in inhibiting Th2 cytokines such as IL-4, with additional effects observed in the suppression of Th1 cytokines, including IFN-γ. Tac-sEVs showed a similar trend in reducing IFN-γ levels, though they do not predominantly target Th1 responses. The mild effect of both treatments on IFN-γ suggests that while they may contribute to managing autoimmune skin disorders associated with elevated IFN-γ levels, their primary action lies in the amelioration of Th2-driven inflammation. Overall, the observed slight reduction in IFN-γ expression from Tacrolimus and Tac-sEVs underlines a potential yet limited capacity to compromise Th1-mediated inflammation. The study demonstrates that SARS-CoV-2 glycoprotein significantly induces IL-4 and IFN-γ production, while tacrolimus and Tac-sEVs effectively suppress these cytokines. The findings underscore the potential of tacrolimus and its sEVs formulation as therapeutic agents for managing inflammatory responses in viral infections, particularly in the context of long COVID.

All data are presented as mean ± standard deviation (SD) from three independent replicates (*n* = 3). Statistical analysis was performed using GraphPad Prism (version X). Two-way analysis of variance (ANOVA) was employed to evaluate the effects of treatment and cytokine type, as well as their interaction. Moreover, the doses of sEVs and Tac-sEVs used in this study were confirmed to be safe for the cells. This was demonstrated through cell toxicity and proliferation studies, where Alamar Blue and CCK-8 assays showed no significant cytotoxic effects and maintained normal cell viability and proliferative capacity at the tested concentrations.

### Ex vivo studies

#### Skin permeation study

**Fig. 4:**
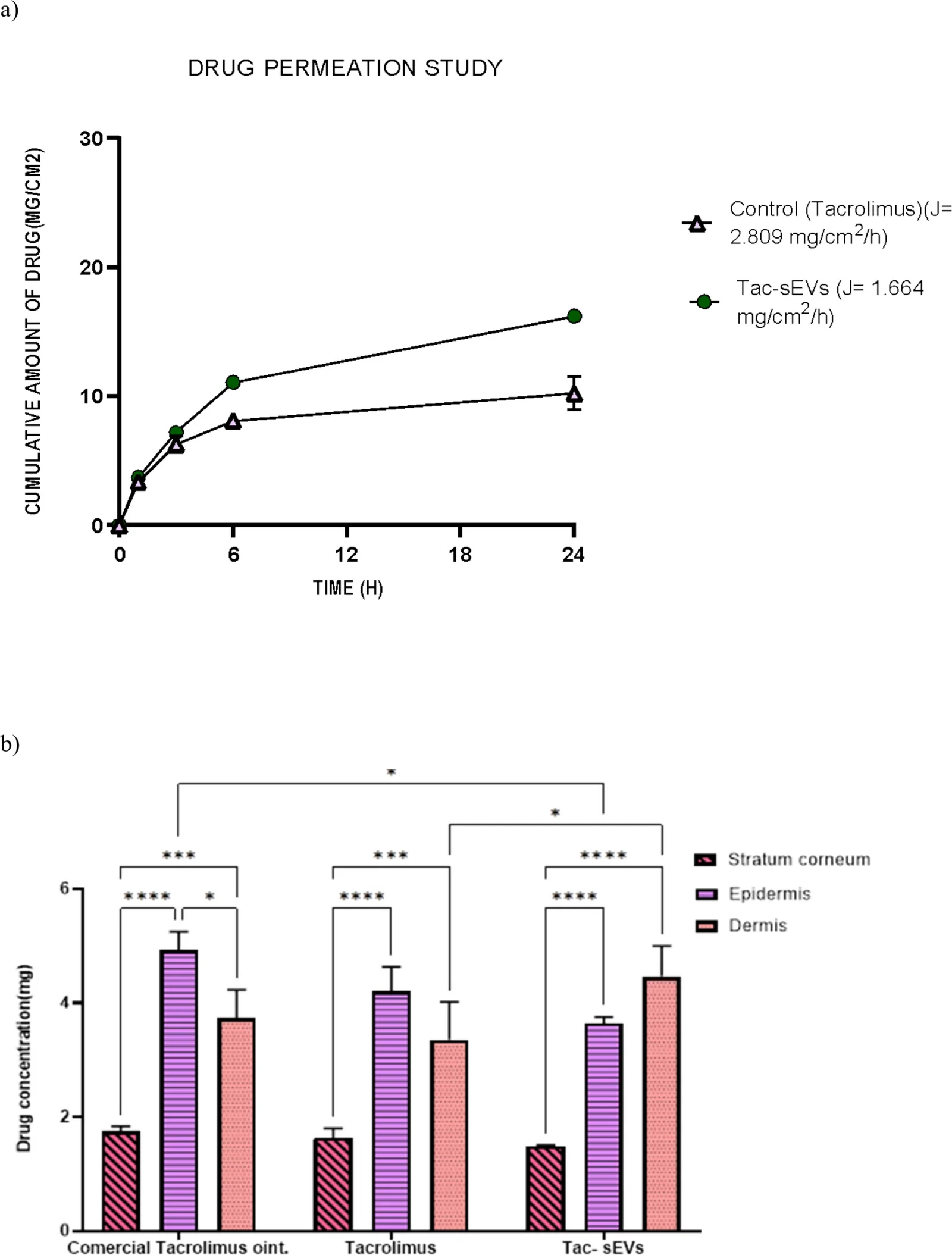
**a** Skin permeation study of tacrolimus and Tacrolimus loaded small extracellular vesicles (Tac-sEVs) by Franz diffusion over 24 hours on porcine skin; *n*=3 ; **b** Dermal pharmacokinetic study by tape stripping on porcine skin with sample size of 3; ****p < 0.0001, ***0.0001 ≤ *p* ≤0.001, *0.01 < *p* < 0.1; *n*=3

The Franz diffusion studies was conducted to rate and extent of drug release from the vesicular formulation and can be used to evaluate the potential of these formulations for topical or transdermal drug delivery. The study (Fig. 4a) shows Tacrolimus being delivered via two distinct formulations, which are free tacrolimus and Tac-sEVs demonstrates the potential advantages of sEVs delivery systems in term of drug permeation and retention through the skin. In the initial permeation phase (0–6 Hours) of the diffusion study, there is an increase in drug permeation for both formulations. This similar release rate at early phase (< 4 h) indicates there were permeation of tacrolimus across the skin potentially due to the lipophilicity of tacrolimus and their interaction with the skin's lipid structures. As the study progresses into the steady state (5–24 h), the cumulative release of drug from Tac-sEVs were significant lower (Flux J = 1.664 mg/cm^2^/h, *p* < 0.05) and much controlled compared to the tacrolimus (Flux J = 2.809 mg/cm^2^/h). This sustained release is indicative of a controlled release mechanism characteristic of sEVs formulation. Tac-sEVs demonstrate an ongoing release profile due to their ability to retain drug molecules and release them gradually, benefiting from their biocompatibility and structural integrity.

The enhanced permeation associated with Tac-sEVs can be explained by their biomechanical characteristics. Small EVs, typically ranging from 100–200 nm, possess a size advantageous for penetrating through the stratum corneum, a vital barrier for transdermal drug delivery. The lipid bilayer of sEVs may facilitate fusion with skin cell membranes, enabling effective release into the skin's deeper layers [54]. The lipid bilayer of sEVs may facilitate fusion with skin cell membranes, enabling effective release into the skin's deeper layers. The findings indicate that Tac-sEVs provide improved drug permeation through the skin compared to free Tacrolimus. The permeation increase seen with Tac-sEVs necessitates a thorough investigation into their potential as nanocarriers. Moreover, the sustained release profile observed with Tac-sEVs, retaining efficacy over a 24-h period, corroborates findings from Chen et al., who noted exosomes' capability for prolonged drug release due to their properties that prevent rapid clearance from biological systems [55]. This prolonged drug retention is crucial for treating chronic conditions that require continuous medication application. Evidence from Bunggulawa et al. indicates that the engineered nature of exosomes facilitates their manipulation to carry specific proteins and drugs over extended periods[56], reinforcing the sustained action observed with Tac-sEVs.

Tacrolimus is a highly lipophilic compound with limited aqueous solubility. The use of PBS as a receptor medium in ex vivo skin permeation studies of tacrolimus is supported by published literature. For instance, Goebel and co-workers employed PBS as the receptor phase in Franz diffusion cell studies to investigate the skin penetration behaviour of tacrolimus formulations, demonstrating that PBS can be suitably applied for comparative permeation assessment of this lipophilic drug [24]. Although surfactants can enhance drug solubility and improve sink conditions, the addition of surfactants may adversely affect the structural integrity and colloidal stability of sEVs by inducing lipid-protein membrane destabilization, vesicle rupture, or premature drug release [57]. Surfactant-containing receptor media were intentionally avoided to prevent confounding effects on vesicle stability and drug release kinetics. To mitigate the limitations associated with PBS, sink conditions were supported by maintaining continuous stirring at 37 °C and by replacing withdrawn receptor samples with fresh PBS at each sampling time point.

#### Ex vivo dermal pharmacokinetics study

Tacrolimus is a highly lipophilic compound with poor aqueous solubility. Therefore, organic solvents such as methanol are employed to extract drug retained. In this work, methanol was used exclusively during the post-experimental extraction step to solubilize tacrolimus retained within skin layers and tape strips. The aim of this study is to identify which formulation of tacrolimus could possibly penetrate deeper into porcine skin layers to effectively treat inflammatory skin conditions. Stratum corneum is the outermost layer of the skin structure. The Fig. 4b below shows statistical analysis of dermal pharmacokinetic study by tape stripping on porcine skin, comparison between free tacrolimus, Tac-sEVs and commercial tacrolimus ointment. All three Tacrolimus formulations demonstrated comparable retention in stratum corneum, with values ranging approximately 1.5 to 1.8 mg. No statistically significant differences were observed between the formulations (*p* > 0.1), indicating that initial drug deposition on the skin surface was similar regardless of formulation. The lack of significant differences indicates that initial deposition of drug on the skin surface is similar across the formulations. However, the initial penetration does not correlate with the formulation’s ability to penetrate and distribute into deeper skin layer. Then the following layer is epidermal layer. The drug concentration in the epidermal layer revealed that commercial Tacrolimus ointment achieved the highest concentration of approximately 4.8 mg. Free Tacrolimus was slightly lower at about 4.2 mg, while Tac sEVs displayed a comparable concentration of around 4 mg. Statistical analysis revealed that the commercial formulation showed a modest but significant increase compared with Tac-sEVs (*p* < 0.1), while no significant difference was observed between free tacrolimus and Tac-sEVs (*p* > 0.1). Although these differences are minimal, they suggest that free Tacrolimus and Tac-sEVs are effective at penetrating the viable epidermis, although commercial formulation shows higher penetration.

In contrast, pronounced and highly significant differences were observed in the dermal layer, the deepest skin compartment examined. Tac-sEVs achieved the highest dermal tacrolimus concentration (~ 4.5 mg), which was significantly greater than both free tacrolimus and the commercial ointment (** *p* < 0.0001). Free tacrolimus demonstrated intermediate dermal penetration (~ 3.6 mg), whereas the commercial formulation exhibited the lowest dermal concentration (~ 3.4 mg). No statistically significant difference was observed between free tacrolimus and the commercial formulation (*p* > 0.1). These results statistically validate the superior dermal delivery capability of Tac-sEVs.

In long COVID, cytokine overdrive can trigger persistent inflammation in the dermis layer, and formulations enabling deeper skin penetration of tacrolimus may help suppress these exaggerated immune responses. The tape stripping results showed that Tac-sEV may be beneficial when it exerts its effects predominantly at the dermal layer, where inflammatory responses are mediated. The dermis harbours various inflammatory cells, including T lymphocytes, which are responsible for the production of pro-inflammatory cytokines. Thus, with the highest concentrations of Tac-sEVs in the dermis, emerge as a promising formulation, potentially improving treatment outcomes for inflammatory skin diseases. The commercial formulation's inferior performance at the dermal layer may limit its effectiveness against conditions requiring deep tissue action.

The findings from this study underscore the importance of developing innovative drug delivery systems that can effectively target the dermis while minimizing systemic side effects. The use of Tac-sEVs, along with other advanced formulations, represents a significant advancement in transdermal drug delivery technology. These approaches not only enhance drug efficacy but also contribute to safer treatment options for patients, reducing the risk of adverse systemic reactions associated with traditional formulations of Tacrolimus.

### Cellular uptake by human dermal fibroblast cells

**Fig. 5:**
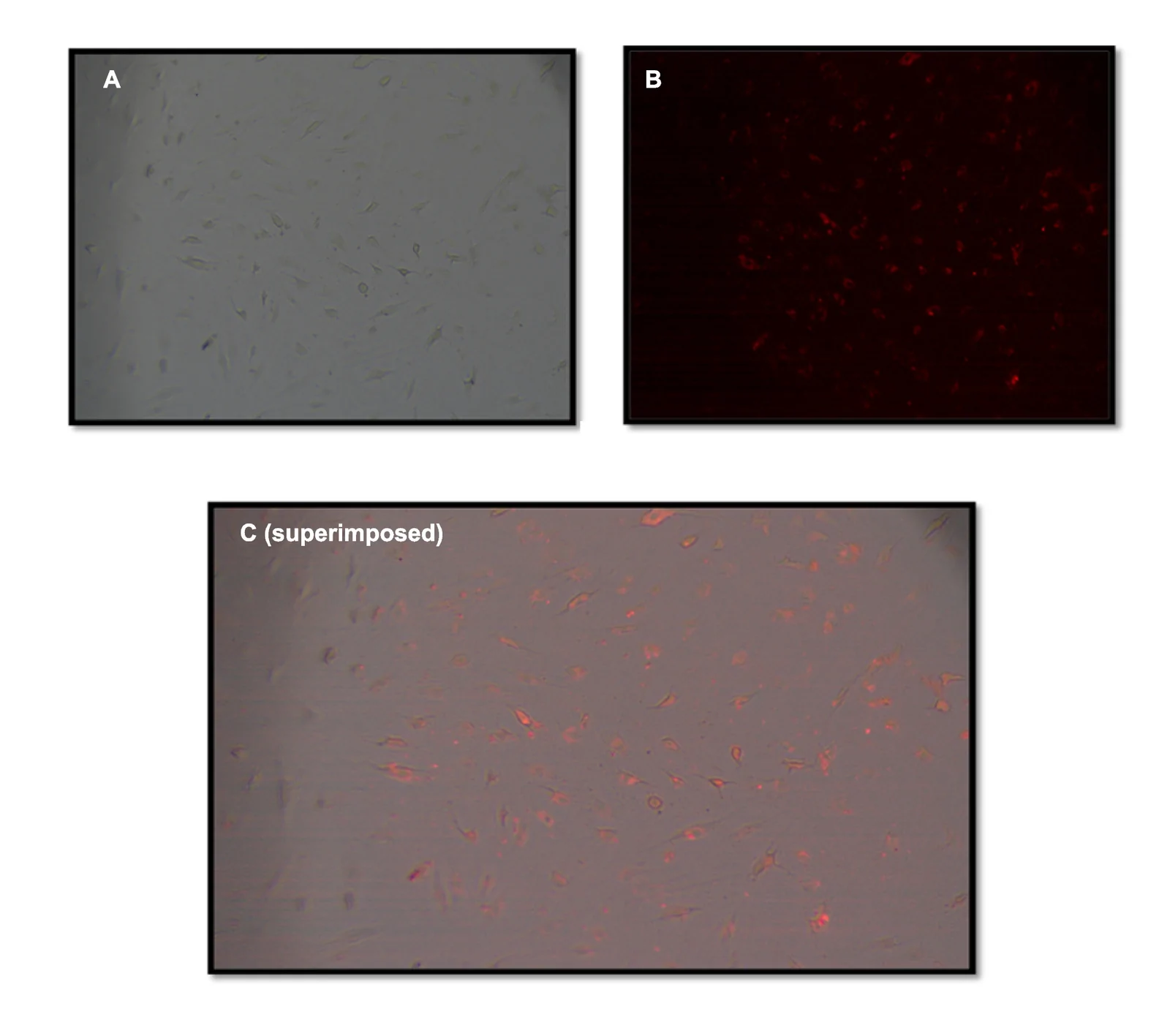
Cellular uptake studies by PKH 26 as fluorescence labelling dye in dermal fibroblast cells under inverted fluorescence microscope (10X magnification). **A** Fibroblast cell; **B** Labelled small extracellular vesicles (sEVs); **C** Superimposed image of cells and labelled sEVs; *n*=3

Figure 5 below shows cellular uptake studies by PKH 26 as fluorescence labelling dye in dermal fibroblast cells under inverted fluorescence microscope. Figure 5(A) shows the image of fibroblast cells, showing the natural morphology of the cells with a spindle-like shape and uniform distribution across the field. Figure 5(B) is the fluorescence image showing PKH26-labelled sEVs. The red fluorescence signal indicates the successful labeling of sEVs, which are visible as numerous red fluorescent puncta spread over the image. The signal is more prominent inside cell boundaries, indicating cellular interaction or uptake. Figure 5(C) is the superimposed image of phase-contrast (A) and fluorescence (B), clearly illustrating internalization of sEVs within the fibroblast cytoplasm. The red fluorescence co localized with the cell structure confirms that the labelled vesicles have been taken up by the cells rather than merely adhering to the surface Fig. 5(C).

**Fig. 6:**
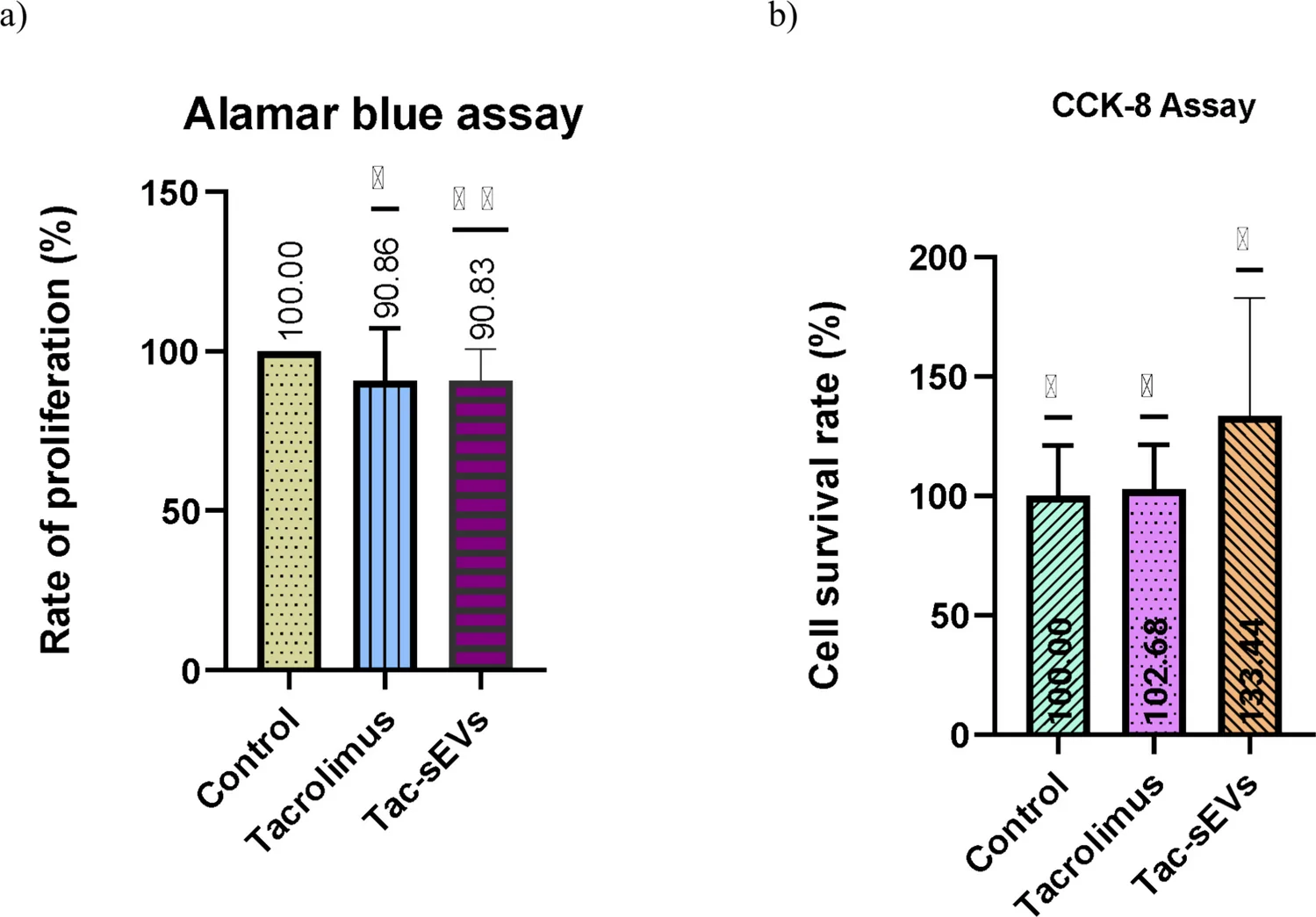
**a** Alamar blue assay and **b** CCK-8 assay of control, Tacrolimus and Tac-sEVs on fibroblast cells where sample size = 3, *p= 0.01, **p=0.004

The analysis of cellular uptake of sEVs labelled with PKH26 dye in fibroblast cells provides significant insights into the mechanisms of vesicle internalization and their potential applications in therapeutic contexts. The observed red fluorescence, indicative of successful internalization, suggests that the sEVs have effectively penetrated the fibroblast cells. This is consistent with previous studies that have demonstrated the capacity of sEVs to be internalized by various cell types through endocytic pathways, including macropinocytosis and receptor-mediated endocytosis [58]. The distribution of fluorescence across the cytoplasm of the fibroblasts further supports the notion that these vesicles are not merely adhering to the cell surface but are actively being taken up and localized within the cellular environment [59].

The efficiency of uptake, as evidenced by the widespread fluorescence among the cell population, indicates a robust interaction between the sEVs and fibroblast cells. This could be attributed to the biological affinity between the source of the sEVs and the fibroblasts, which may enhance the likelihood of successful internalization [60]. Such interactions are critical for the therapeutic potential of sEVs, as they are increasingly being recognized as effective carriers for drug delivery and intercellular communication. The punctate nature of the fluorescence observed within the cells may suggest the presence of endosomal compartments, which is a common characteristic of vesicle uptake and processing [61]. The integrity of the PKH26 signal is also noteworthy, as minimal background fluorescence outside the cells implies that proper washing steps were employed post incubation. This is crucial for ensuring that the observed fluorescence is indeed due to the internalized sEVs and not residual unbound dye, which could confound the results. The effective removal of unbound dye enhances the reliability of the findings and supports the conclusion that the observed fluorescence is a direct result of sEV uptake.

The implications of these findings are significant, particularly in the context of therapeutic applications. The successful internalization of sEVs into fibroblast cells underscores their potential as vehicles for delivering therapeutic agents, such as RNA, proteins, or small molecules, directly to target cells. This capability is particularly relevant in regenerative medicine, drug delivery and cancer therapy, where sEVs can be engineered to carry specific therapeutic payloads that can modulate cellular responses or promote tissue repair. Furthermore, understanding the mechanims of sEVs uptake can inform the design of more effective delivery systems, enhancing the efficacy of treatments that utilize these vesicles.

## Conclusion

This study has successfully demonstrated the potential of Tac-sEVs as a promising drug delivery system to manage cytokine-driven inflammatory skin conditions. The characterization of sEVs confirmed successful isolation using sucrose cushion ultracentrifugation method. Transmission Electron Microscopy (TEM) revealed their spherical morphology, while Nanoparticle Tracking Analysis (NTA) confirmed their nanoscale size and uniform distribution. Dot blot analysis confirmed the presence of sEV markers (CD63, HSP70, TSG101), and protein quantification using the MicroBCA assay further validated their successful isolation. Following tacrolimus encapsulation, Tac-sEVs were characterized for their morphology, size, encapsulation efficiency, and drug release profile. TEM and NTA confirmed that Tac-sEVs retained the morphology and size of sEVs, ensuring their stability as drug carriers. The encapsulation efficiency of Tac-sEVs was recorded at 79.19% ± 0.002, demonstrate the effective drug loading. The in vitro studies confirmed that Tac-sEVs significantly suppress the expression of pro-inflammatory cytokines, including IFN-γ, GCS-F, IL-2, and IL-4, demonstrating their immunosuppressive properties. Moreover, ex vivo studies using Franz diffusion and tape stripping techniques revealed that Tac-sEVs penetrated deeper into dermal layer via controlled release manner. Cellular uptake studies further confirmed the successful internalization of sEVs by human dermal fibroblast cells, supporting their role in targeted drug transport. Additionally, cytotoxicity assessments through Alamar Blue and CCK-8 assays showed high cell viability, confirming the biocompatibility and minimal toxicity of Tac-sEVs. Long COVID–related skin inflammation is often sustained by overproduction of pro-inflammatory cytokines such as IFN-γ, IL-2, IL-4, and GCS-F, leading to recurrent flares and prolonged healing. The present study demonstrates that Tac-sEVs not only penetrate deeper into the dermis—where cytokine activity is most pronounced—but also provide sustained drug release and potent suppression of these key cytokines. Compared to conventional tacrolimus formulations, Tac-sEVs achieved enhanced dermal delivery, improved cellular uptake, and maintained high biocompatibility in human skin cells. These properties directly address the limitations of current therapies, offering a targeted, safer, and potentially more effective approach for managing chronic, cytokine-driven skin manifestations associated with long COVID.

Despite providing valuable insights, this study has several limitations that may affect its clinical translation. The exclusive use of in vitro models, including human dermal fibroblasts and Franz diffusion studies, limits physiological relevance due to the absence of systemic interactions and long-term responses. Additionally, focusing primarily on fibroblasts does not fully capture the complexity of skin inflammation, which involves multiple cell types. The study also demonstrated cytokine suppression without in-depth investigation of underlying signaling pathways such as NF-κB and MAPK. Furthermore, the lack of comparative evaluation with established drug delivery systems restricts assessment of the relative advantages of Tac-sEVs. Therefore, future studies incorporating in vivo models, biological mechanisms of tacrolimus release, and cytokine modulation via sEVs are necessary to strengthen the translational potential of Tac-sEVs for skin inflammation therapy.

## Data Availability

All data generated or analyzed during this study are included in this published article and its Supplementary information files and raw data are available from the corresponding author upon reasonable request.
